# Relativistic Diffusing Particles, Entropy and Varying Cosmological Constants

**DOI:** 10.3390/e28101152

**Published:** 2026-10-20

**Authors:** Zbigniew Haba

**Affiliations:** Institute of Theoretical Physics, University of Wroclaw, Plac Maxa Borna 9, 50-204 Wroclaw, Poland; zbigniew.haba@uwr.edu.pl

**Keywords:** relativistic diffusion, expanding universe, theormodynamics

## Abstract

We discuss Einstein equations with a non-conserved energy-momentum $T^{\mu \nu }$. The energy-momentum tensor ${\tilde{T}}^{\mu \nu }$ on the right hand side of Einstein equations is expressed in the form $8\pi GT^{\mu \nu }-g^{\mu \nu }\Lambda +\Sigma^{\mu \nu }$ where *G* is the Newton constant, Λ is the cosmological term and $T^{\mu \nu }$ is the energy-momentum of an ideal fluid. The remaining part $\Sigma^{\mu \nu }$ describes the viscosity and a heat flow. We discuss space-time-dependent Newton “constant” *G* and the cosmological term Λ. The covariant conservation law for ${\tilde{T}}^{\mu \nu }$ imposes a relation between the energy-momentum (non)conservation and the variation of *G* and Λ. The covariant derivative $\nabla_{\mu }T^{\mu \nu }$ can be related to the first law of thermodynamics and expressed by the temperature *T* and the entropy *S*. The energy-momentum non-conservation in our models is a consequence of a diffusion of its constituting particles. We describe a gas of diffusing massive particles in a homogeneous isotropic space-time as a system with a time-dependent linear barotropic equation of state $P_{t}=w_{t}\rho_{t}$ with $0<w_{t}\leq \frac{1}{3}$. The gas of massive diffusing particles in an expanding universe tends in a limit of infinite time to the gas of diffusing massless particles. We obtain results on the energy density, entropy and phase space dstribution in a soluble model of massless relativistic particles in a spatially flat expanding universe.

## 1. Introduction

The geometry of the universe in Einstein general relativity is determined by the energy-momentum tensor ${\tilde{T}}_{\mu \nu }$ (μ,ν=0,1,2,3, we use the conventions of [1])

(1) $$G_{\mu \nu }={\tilde{T}}_{\mu \nu },$$

where $G_{\mu \nu }=R_{\mu \nu }-\frac{1}{2}g_{\mu \nu }R$ ($R_{\mu \nu }$ is the Ricci tensor). The standard Einstein equations can be expressed in the form (velocity of light c=1)

(2) $$G_{\mu \nu }={\tilde{T}}_{\mu \nu }\equiv 8\pi GT_{\mu \nu }-g_{\mu \nu }\Lambda +\Sigma_{\mu \nu }.$$

*G* is the Newton constant. The tensor $T_{\mu \nu }$ on the rhs of Equation (2) is usually assumed in the form of an ideal fluid. Λ is the cosmological term. $\Sigma_{\mu \nu }$ describes viscosity and a heat flow in the fluid [2,3]. Such a term is usually discarded (see, however, [4]). The system (2) with constant *G* and Λ (without $\Sigma_{\mu \nu }$) constitutes the basis of the standard ΛCDM model. There are some attempts to extend the ΛCDM model (see [5]) to varying cosmological constants in order to explain some problems in this model (the need for dark matter, coincidence problem, $H_{0}$-tension [6]). The coincidence problem could be approached by an assumption that the baryonic matter, dark matter and dark energy are related by an interaction. In refs. [7,8] we discussed a model with a diffusing dark matter. In order to work with a conserved energy-momentum ${\tilde{T}}_{\mu \nu }$ (with constant *G* and Λ) we had to add a compensating energy-momentum for the dark energy.

In this paper we discuss Einstein equations for a system of particles (baryons and dark matter) moving in a passive medium. The non-conserved energy-momentum can appear in Einstein equations if the Newton “constant” and (or) the cosmological term are space-time dependent. We consider Einstein general relativity of a fluid of relativistic particles described by a phase space distribution Ω. The phase space distribution defines the energy-momentum tensor entering the Einstein equation and the particle current applied for a decomposition of the energy-momentum tensor in its ideal fluid and a dissipative part. However, in prospective studies we would apply the phase space distribution in cosmology for a description of formation and dynamics of clusters of galaxies. The energy-momentum tensor is not conserved if some degrees of freedom in Ω are ignored (so that there is just an averaging over these degrees of freedom). We obtain a dissipative system. The Markov approximation leads to a diffusive description of the neglected degrees of freedom. Such a description is well-known from the theory of Brownian motion. The Brownian particles are described in statistical physics by their probability distribution. A detailed knowledge of the neglected degrees of freedom (the medium) are not needed. The medium just contributes to the diffusion constant. The (incomplete) energy-momentum $T^{\mu \nu }$ on the rhs of Einstein Equations (1) and (2) does not contradict Bianchi identities if the Newton “constant” *G* and (or) the cosmological term Λ are space-time dependent. Space-time dependence of *G* and Λ in Einstein equations has been discussed earlier in [9,10,11,12,13,14,15,16,17,18,19,20]. In theoretical models the space-time dependence of cosmological constants can result from an addition of some other fields to the model (as in [9]) or from quantum effects incorporated into the classical model (as vacuum expectation value of the energy-momentum tensor or renormalization of the Newton constant). The variation of the Newton “constant” *G* can also be explained in the phase space picture as a presence of some unobservable particles which interact with the baryonic and dark matter. The fluid ${\tilde{T}}^{\mu \nu }$ may consist of conserved matter (which does not interact with the rest of the system) and the dark matter diffusing in an unspecified medium. The particles of this medium do not enter the energy-momentum ${\tilde{T}}^{\mu \nu }$ but their presence is experienced as a diffusion of the dark matter and a variation of cosmological constants.

Diffusing matter has been introduced in Einstein gravity in some earlier papers. A modification of Einstein gravity by an energy-momentum tensor of a diffusion (resulting from a discreteness of space-time) has been discussed in [21,22,23,24,25]. This model can be considered as a version of unimodular gravity [25] which allows energy-momentum non-conservation if the covariant derivative of the energy-momentum is related to the cosmological term. In our approach, in order to preserve the general covariance we consider the relativistic diffusion. The relativistic diffusion is a well-studied model [26,27,28,29]. Its application to cosmology has been considered in [7,30,31,32,33,34,35,36,37,38,39]. In [21,22,24], the model was applied to an explanation of dark energy and the $H_{0}$ tension. In such a model the dark energy results from a non-conservation of the energy of the diffusive matter. This is a certain modification of the model [7,8,30,37,38,39,40] where the dark energy has its own energy-momentum in order to compensate for the dark energy-momentum non-conservation. The phenomena with a violation of the energy-momentum conservation cannot be described by Einstein equations with fixed cosmological constants. We must either compensate $\nabla_{\mu }T^{\mu \nu }$ by another (non-conserved) energy-momentum of the dark energy or admit varying cosmological constants (a varying Λ in Einstein gravity with a diffusion has been considered in [32]).

In this paper we study varying constants and a non-conservation of the energy-momentum $T^{\mu \nu }$ of an ideal fluid. In Section 2 we obtain the Bianchi identities resulting from a conservation of ${\tilde{T}}^{\mu \nu }$(1) and non-conservation of $T^{\mu \nu }$ (2). A covariant conservation of the total energy-momentum ${\tilde{T}}^{\mu \nu }$ (2) (including the varying *G* and Λ) leads to equations relating the Newton “constant” and (or) the cosmological term Λ to $\nabla_{\mu }T^{\mu \nu }$. In Section 3 we introduce a model of a gas of relativistic diffusing particles in an expanding universe. The thermodynamics of such a gas is discussed in Section 4. In Section 5 we consider a Jüttner-type solution for diffusing relativistic massless particles. The model of massless particles can be considered as a large time limit of the massive one. In this model we study the effect of diffusing particles on the possible variation of *G* and Λ. According to the first law of thermodynamics $\nabla_{\mu }T^{\mu \nu }$ is related to the heat flow. Then owing to the second law of thermodynamics the heat flow should be expressed by the change of entropy TdS (where *T* is the temperature). In Section 5, by applying the thermodynamics, we express the variation of cosmological constants by the entropy *S* of a gas of diffusing particles (in particular, we obtain lnG≃S). In Section 6 we describe the entropy of a diffusing gas in more detail. We show that the microscopic definition of the entropy coincides with the thermodynamic definition. In Section 7 we discuss solutions of the diffusion equation in a fixed metric and subsequently the Einstein equations which determine this metric. In Appendix A, we study in detail the general solution of the massless diffusion equation in an expanding metric. In particular, we discuss the exponential expansion of the universe and the coasting cosmology [41] which appear as distinguished solutions of Einstein and diffusion equations. A solution of the diffusion equation together with Einstein equations describes a distribution of particles (galaxies) in the phase space defined by the universe evolution.

## 2. Einstein Equations and Bianchi Identities

The Bianchi identity applied to Equation (1) leads to the equation

(3) $$\nabla_{\mu }{\tilde{T}}_{\nu }^{\mu }=0.$$

If we can define a time-like fluid four-velocity $u^{\mu }$ (satisfying the normalization $u_{\mu }u^{\mu }=-1$) then we can decompose any energy-momentum tensor in the form [2,3]

(4) $$\begin{array}{c} {\tilde{T}}^{\mu \nu }=8\pi GT^{\mu \nu }+\Sigma^{\mu \nu }-g^{\mu \nu }\Lambda \equiv {\hat{T}}^{\mu \nu }-g^{\mu \nu }\Lambda \end{array}$$

where (ideal fluid)

(5) $$T^{\mu \nu }=\left(\rho +P\right)u^{\mu }u^{\nu }+g^{\mu \nu }P.$$

Then, ρ has the meaning of the energy-density and *P* is the pressure.

We can express ρ, *P* and $\Sigma^{\mu \nu }$ using suitable projections of the energy-momentum tensor ${\tilde{T}}^{\mu \nu }$ of Equation (4). These projections give

(6) $$8\pi G\rho +\Lambda =u^{\mu }{\tilde{T}}_{\mu \nu }u^{\nu },$$

(7) $$8\pi GP-\Lambda =\frac{1}{3}{\tilde{T}}_{\mu \nu }{▵}^{\mu \nu }$$

with

(8) $${▵}_{\alpha }^{\mu }=\delta_{\alpha }^{\mu }+u^{\mu }u_{\alpha }.$$

Then,

(9) $$\Sigma^{\mu \nu }=I^{\mu }u^{\nu }+I^{\nu }u^{\mu }+{\tilde{\Pi }}^{\mu \nu }$$

$I^{\mu }$ has the meaning of the energy flow. It is subtracted from $\Sigma^{\mu \nu }$ as

(10) $$I^{\mu }=u_{\nu }{\tilde{T}}^{\alpha \nu }{▵}_{\alpha }^{\mu }.$$

where the tension tensor ${\tilde{\Pi }}^{\mu \nu }$ is separated from $\Sigma^{\mu \nu }$ by the condition

(11) $${\tilde{\Pi }}^{\mu \nu }u_{\mu }={\tilde{\Pi }}^{\mu \nu }g_{\mu \nu }=0.$$

The Bianchi identity (3) gives an equation for the “fluids”

(12) $$8\pi \partial_{\mu }GT_{\nu }^{\mu }+8\pi G\nabla_{\mu }T_{\nu }^{\mu }-\partial_{\nu }\Lambda +\nabla_{\mu }\Sigma_{\nu }^{\mu }=0.$$

We decompose the Bianchi identities (12) into the parts parallel to the velocity (through a multiplication of Equation (12) by $u^{\mu }$) and orthogonal to the velocity (through a multiplication by ${▵}_{\mu \alpha }$). The parallel part is

(13) $$\begin{array}{c} -8\pi u^{\mu }\partial_{\mu }G\rho -8\pi Gu^{\mu }\partial_{\mu }\rho -u^{\nu }\partial_{\nu }\Lambda -8\pi G\left(\rho +P\right)\nabla_{\mu }u^{\mu }+u_{\nu }\nabla_{\mu }\Sigma^{\mu \nu }=0 \end{array}$$

The orthogonal part reads

(14) $$\begin{array}{c} 8\pi P\partial^{\mu }G{▵}_{\mu \alpha }+8\pi G\partial^{\mu }P{▵}_{\mu \alpha }-{▵}_{\nu \alpha }\partial^{\nu }\Lambda +8\pi G\left(\rho +P\right)u^{\mu }\nabla_{\mu }u^{\nu }{▵}_{\nu \alpha }\\ +{▵}_{\nu \alpha }\nabla_{\mu }\Sigma^{\mu \nu }=0. \end{array}$$

In general, it is difficult to solve the Bianchi identities. Note, however, that if $\partial_{j}P=\partial_{j}G=\partial_{j}\Lambda =0$ then in the comoving frame the orthogonal Bianchi identities are satisfied. In the next section we discuss relativistic systems with a phase space distribution Ω(x,p). If the dependence of Ω on the momentum *p* is in the form $p_{\mu }u^{\mu }$ with a certain four-vector *u* then the energy-momentum is in the form of an ideal fluid (5) (for a general phase space distribution it will have the form (4)).

We assume that the Riemannian manifold is globally hyperbolic. Then, we can introduce coordinates [42] such that the metric takes the form

(15) $$dl^{2}=g_{\mu \nu }dx^{\mu }dx^{\nu }=g_{00}dt^{2}+\gamma_{jk}dx^{j}dx^{k}=-\left|g_{00}\right|dt^{2}+\gamma_{jk}dx^{j}dx^{k}.$$

We shall discuss models with a particle current (a stream of particles) $N^{\mu }$. Such a current exists if the particles have a phase space distribution (Section 3). Then, Eckart’s [43] definition of the fluid velocity is

(16) $$u^{\mu }={\left(-N_{\nu }N^{\nu }\right)}^{-\frac{1}{2}}N^{\mu }.$$

A solution of Einstein equations describing an expanding isotropic homogeneous universe (with the curvature index k=−1,0,1) has the FLRW metric

(17) $$dl^{2}=-dt^{2}+a^{2}\left({\left(1-kr^{2}\right)}^{-1}dr^{2}+r^{2}\left(d\theta^{2}+\sin^{2}\theta d\phi^{2}\right)\right).$$

In the metric (17) the fluid velocity satisfies $\nabla_{\mu }u^{\mu }={\left(-g\right)}^{-\frac{1}{2}}\partial_{\mu }\left({\left(-g\right)}^{\frac{1}{2}}u^{\mu }\right)=3H$ (where $g=\det(g_{\mu \nu })$ and $H=a^{-1}\partial_{t}a$). For the ideal fluid we have

(18) $$u^{\nu }\nabla_{\mu }T_{\nu }^{\mu }=-\left(\rho +P\right)\nabla_{\mu }u^{\mu }+u^{\mu }\nabla_{\mu }\rho .$$

Hence, from Equations (13) and (18) under the assumption that ρ and *p* depend only on time,

(19) $$8\pi \partial_{t}\left(G\rho \right)+24\pi GH\left(\rho +P\right))+\partial_{t}\Lambda =0$$

If P=wρ then Equation (19) is an equation for Gρ. However, if the relation between ρ and *P* is non-linear (e.g., a polytropic gas) then *G* and ρ enter separately Einstein equations and Bianchi identities.

$T^{\mu \nu }$ may be a sum of many fluids with some conserved parts in it

(20) $$\rho =\rho^{c}+\tilde{\rho },$$

where by the index “*c*” we mean a fluid with a conserved energy-momentum

(21) $$\partial_{t}\rho^{c}+3H\left(\rho^{c}+P^{c}\right)=0.$$

If $\tilde{P}=\tilde{w}\tilde{\rho }$ and $P^{c}=w^{c}\rho^{c}$ then Equation (19) depends separately on ρ and *G*

(22) $$8\pi \partial_{t}\left(G\rho \right)+24\pi H\left(1+w^{c}\right)G\rho^{c}+24\pi H\left(1+\tilde{w}\right)G\left(\rho -\rho^{c}\right)+\partial_{t}\Lambda =0$$

Equation (14) is satisfied if *P* depends only on time as (in the comoving frame u=(1,0)), $T^{0j}=0$ and $\nabla_{j}T_{k}^{j}=\partial_{k}P=0$.

The Einstein Equation (1) in the metric (17) can be decomposed in the comoving frame in the 00 and μj parts. The $G^{00}$ part is

(23) $${\left(a^{-1}\frac{da}{dt}\right)}^{2}=-\frac{k}{a^{2}}+G\frac{8\pi }{3}\rho +\frac{1}{3}\Lambda .$$

Adding the $G^{00}$ equation to the $G^{kj}$ equation we obtain the second (Friedmann) equation

(24) $$a^{-1}\frac{d^{2}a}{dt^{2}}=-G\frac{4\pi }{3}\left(\rho +3P\right)+\frac{1}{3}\Lambda .$$

The second Friedmann Equation (24) follows from the first Equation (23) and the conservation law (19) (even if *G* and Λ are time dependent).

## 3. Non-Conserved Energy-Momentum: Relativistic Diffusion

In this section we consider a gas of particles moving on a (pseudo)Riemannian manifold and described by a phase space distribution Ω. We concentrate on a model of relativistic particles diffusing in a medium expanding in the metric (15). Let us briefly review the model which may have a wider application in astrophysics and cosmology [7,8,21,22,23,28,30,32,33,35,36]. A gas of particles is described by a distribution Ω on the phase space [44,45,46,47]. If the particle’s dynamics (geodesics) are determined by classical evolution equations in a gravitational field, then the energy-momentum conservation law is a consequence of the Liouville equation [44] (where $\Gamma_{\nu \rho }^{\mu }$ are the Christoffel symbols)

(25) $$\begin{array}{c} (p^{\mu }\partial_{\mu }^{x}-\Gamma_{\mu \nu }^{k}p^{\mu }p^{\nu }\partial_{k}^{p})\Omega =0, \end{array}$$

where $\partial^{p}$ denotes derivatives over the momenta and $\partial^{x}$ is a derivative over a space-time coordinate *x*. The Greek indices run from 0 to 3; Latin indices denoting spatial components have the range from 1 to 3. The energy-momentum tensor is defined by

(26) $${\hat{T}}^{\mu \nu }=\sqrt{g}\int dpp_{0}^{-1}p^{\mu }p^{\nu }\Omega ,$$

where $p_{0}$ is determined from the mass-shell condition $p_{\mu }p^{\mu }=-m^{2}$ (*m* is the particle’s mass). We can also define the conserved particle current ($\nabla_{\mu }^{x}N^{\mu }=0$)

(27) $$N^{\mu }=\sqrt{g}\int dpp_{0}^{-1}p^{\mu }\Omega .$$

${\hat{T}}^{\mu \nu }$ in general is not of the form of an ideal fluid (5) but has a decomposition (4)–(9). However, in subsequent sections we shall consider solutions of the diffusion equation which lead to the energy-momentum tensor of the form of the ideal fluid (5).

We could consider a more general phase space distribution

$${\hat{\Omega }}_{t}\left(x,p,l\right)=\Omega_{t}\left(x,p\right)\omega_{t}\left(x,l\right)$$

describing different sets of particles with momenta p and l. If we do not include the $l^{\mu }l^{\nu }$ integrals of Equation (26) into the energy-momentum tensor ${\tilde{T}}^{\mu \nu }$ for the phase space distribution ${\hat{\Omega }}_{t}$ then their sole effect is the multiplicative factor $G\to G\int dl\omega_{t}\left(x,l\right)$ in the energy-momentum tensor (26). In general, without the inclusion of the l- particles the energy-momentum tensor (26) will be not conserved.

We preserve the Formulas (26) and (27) for a diffusion which still is described by a phase space distribution $\Omega_{t}\left(x,p\right)$. The diffusion can be considered as a particle motion along random geodesics. The diffusion takes place on the hyperboloid $H_{+}$ (the positive energy mass-shell; see [26,27,28,30,33]). $N^{\mu }$ (27) is a time-like vector which can be related to a fluid velocity current $N^{\mu }=Nu^{\mu }$ according to Equation (16). The transport equation is

(28) $$\begin{array}{c} \left(p^{\mu }\partial_{\mu }^{x}-\Gamma_{\mu \nu }^{k}p^{\mu }p^{\nu }\partial_{k}^{p}\right)\Omega =\kappa^{2}{▵}_{H}\Omega , \end{array}$$

where $\kappa^{2}$ is the diffusion constant.

The operator ${▵}_{H}$ is the Laplace–Beltrami operator on the mass hyperboloid $H_{+}$

$$g_{00}p^{0}p^{0}+\gamma_{jk}p^{j}p^{k}=-m^{2}.$$

The metric is defined by

(29) $$ds^{2}=m^{-2}g_{\mu \nu }dp^{\mu }dp^{\nu }\equiv h_{jk}dp^{j}dp^{k},$$

where $p^{0}$ is expressed by spatial components

(30) $$p^{0}=\sqrt{|g_{00}{|}^{-1}\left(m^{2}+\gamma_{jk}p^{j}p^{k}\right)}.$$

The metric is

(31) $$h_{jk}=m^{-2}\left(\gamma_{jk}-p_{j}p_{k}\omega_{g}^{-2}\right),$$

where $p_{j}=\gamma_{jk}p^{k}$ and

(32) $$\omega_{g}^{2}=m^{2}+\gamma_{jk}p^{j}p^{k}.$$

Then

(33) $${▵}_{H}=\frac{1}{\sqrt{h}}\partial_{j}^{p}h^{jk}\sqrt{h}\partial_{k}^{p},$$

where

(34) $$h^{jk}=m^{2}\gamma^{jk}+p^{j}p^{k},$$

$\partial_{j}^{p}=\frac{\partial }{\partial p^{j}}$ and $h=\det\left(h_{jk}\right)=m^{-4}\det\left(\gamma_{jk}\right)\omega_{g}^{-2}$ is the determinant of the metric $h_{jk}$.

If κ≠0 then the energy-momentum tensor is not conserved but [33,34]

(35) $$\nabla_{\mu }^{x}{\hat{T}}^{\mu \nu }=3\kappa^{2}N^{\nu }.$$

Now Equation (12) reads (where $T^{\mu \nu }\to {\hat{T}}^{\mu \nu }$)

(36) $$8\pi \partial_{\mu }G{\hat{T}}_{\nu }^{\mu }+24\pi G\kappa^{2}N_{\nu }-\partial_{\nu }\Lambda =0.$$

In our model the particle current is conserved (as a consequence of Equation (28))

(37) $$\nabla_{\mu }N^{\mu }={\left(-g\right)}^{-\frac{1}{2}}\partial_{\mu }\left({\left(-g\right)}^{\frac{1}{2}}N^{\mu }\right)=0.$$

This means that the particle number is constant

(38) $$\begin{array}{c} \partial_{t}\int dx\left({\left(-g\right)}^{\frac{1}{2}}N^{0}\right)=-\int dx\partial_{j}{\left(-g\right)}^{\frac{1}{2}}N^{j})=0 \end{array}$$

In the comoving frame only the temporal component of the current is not zero

(39) $$N^{0}=n{\left(-g\right)}^{-\frac{1}{2}},$$

where *n* may be space dependent.

The zeroth component of Equation (36) reads (assuming *G* and Λ depend only on time)

(40) $$8\pi \partial_{t}G{\hat{T}}_{0}^{0}+24\pi G\kappa^{2}ng_{00}{\left(-g\right)}^{-\frac{1}{2}}-\partial_{t}\Lambda =0.$$

From now on we restrict ourselves to a flat expanding metric (k=0 in FLRW metric (17))

(41) $$dl^{2}=-dt^{2}+a^{2}dx^{2}$$

Writing $\nabla_{\mu }{\hat{T}}_{0}^{\mu }=3\kappa^{2}N_{0}$ in terms of ρ and *P* we obtain

(42) $$\partial_{t}\rho +3H\left(\rho +P\right)=3\kappa^{2}n{\left(-g\right)}^{-\frac{1}{2}}.$$

This equation can be solved for ρ if P=wρ. Then, we can express the solution of Equation (42) as

(43) $$\begin{array}{c} \rho \left(t\right)=a^{-3(1+w)}\left(\rho \left(t_{0}\right)a{\left(t_{0}\right)}^{3(1+w)}+3n\kappa^{2}\int_{t_{0}}^{t}a{\left(s\right)}^{3w}ds\right)\\ =3na^{-3(1+w)}\left(T_{0}+\kappa^{2}\int_{t_{0}}^{t}a{\left(s\right)}^{3w}ds\right), \end{array}$$

where we have defined the initial conditions by means of a parameter $T_{0}$ (which will be related to the initial or present time temperature at $t_{0}$, see Section 4) in such a way that

$$\rho \left(t_{0}\right)=3na_{0}^{-3(1+w)}T_{0}.$$

In a way similar to the standard (non-relativistic) Brownian motion with a friction we can ascribe a temperature *T* to the gas of diffusing particles [48]. The formula for the temperature follows from Equation (43) ([8] and Section 4, Equation (62))

(44) $$T=a^{-3w}\left(T_{0}+\kappa^{2}A\right).$$

where

$$A=\int_{t_{0}}^{t}a{\left(s\right)}^{3w}ds.$$

Note that

$$\frac{dT}{dt}=-3wHT+\kappa^{2}.$$

For an exponential expansion a=exp(Ht) we have

$$T=\exp\left(-3wHt\right)\left(T_{0}-\frac{\kappa^{2}}{3wH}\exp\left(3wHt_{0}\right)\right)+\frac{\kappa^{2}}{3wH}$$

For a power-law expansion $a=t^{\alpha }$ we get

$$\begin{array}{c} T=\left(T_{0}-\frac{\kappa^{2}}{1+3w\alpha }t_{0}^{1+3w\alpha }\right)t^{-3w\alpha }+\frac{\kappa^{2}}{1+3w\alpha }t. \end{array}$$

Hence, for an exponential expansion the temperature of the fluid is decaying (from its value $\exp\left(-3Hwt_{0}\right)T_{0}$), tending to a constant with the speed $a^{-3w}$ when t→∞. For a power-law expansion it is decaying from $T_{0}t_{0}^{-3w\alpha }$ at small $t\geq t_{0}$ with the rate $a^{-3w}$ (in agreement with the adiabatic thermodynamics of Section 4) and subsequently linearly growing to infinity for a large time.

Let us consider now the universe evolution determined by Equation (41) and the Bianchi identity resulting from Equations (12) and (40)

(45) $$a^{-3(1+w)}\left(T_{0}+\kappa^{2}A\right)\partial_{t}G+G\kappa^{2}a^{-3}=-\frac{1}{24n\pi }\partial_{t}\Lambda$$

If G=const then the Bianchi identity (45), the expression of the energy-momentum non-conservation (42) and the first Friedmann Equation (23) constitute a closed system of first order differential equations for Λ,ρ,a with the unique solution under given initial conditions (in particular, we may assume for Λ zero or non-zero initial condition). If G=const then it follows (this is an equation of Perez et al. [21] and Calogero [32])

(46) $$\Lambda =\Lambda_{ex}-24\pi nG\kappa^{2}\int_{t_{0}}^{t}a^{-3}$$

When we insert ρ (43) and Λ (46) at G=const in the first Friedman Equation (23) then we obtain an integro-differential equation which is difficult to solve exactly. At small $\kappa^{2}$ we have the standard (non-diffusive) behaviour of the solutions of the Friedmann equations. $\Lambda_{ex}$ in our model is just an arbitrary parameter in the initial condition. If $\Lambda_{ex}>0$ then the Friedmann equations will have solutions with the asymptotic behaviour $a≃\exp(t\sqrt{\frac{\Lambda_{ex}}{3}})$. If $\Lambda_{ex}=0$ then asymptotically a≃t at large time (coasting cosmology [41]). This is different from $t^{\frac{2}{3(1+w)}}$ for the evolution without diffusion. If we admit *G* to depend on time, then in order to have the unique solution we must fix one of the functions (possibly from astronomical observations) *a*, ρ, Λ or *G* (this still will be discussed in Section 7).

If Λ=const and $\rho^{c}=0$ (under the assumption P=wρ) then the solution of Equation (22) is $G\rho =Ka^{-3(1+w)}$. Hence, the diffusion has no effect on the first Friedmann equation (we obtain the standard solution for the Friedmann equation). However, according to Equation (45) (owing to the additional Equation (35))

(47) $$G=G_{0}T_{0}{\left(T_{0}+\kappa^{2}A\right)}^{-1}.$$

If there is to be a modification of the gravitational potential resulting from the source Gρ then we must assume a varying Λ. In general, from Equation (45)

(48) $$\begin{array}{c} G=G_{0}T_{0}{\left(T_{0}+\kappa^{2}A\right)}^{-1}-\frac{1}{24\pi n}{\left(T_{0}+\kappa^{2}A\right)}^{-1}\int_{t_{0}}^{t}a^{3(1+w)}\partial_{s}\Lambda ds. \end{array}$$

The solution Ω of the diffusion equation defines ρ and *P* as a function of *a*. The Friedmann Equation (23) determines *a* as a function of *G* and Λ. Then, the Bianchi identity (19) relates *G* to Λ.

Let us consider the diffusion Equation (28) in more detail in the spatially flat FLRW model (k=0 in Equation (17)). In the metric (41) the non-vanishing Christoffel symbols are

$$\Gamma_{jk}^{0}=a\frac{da}{dt}\delta_{jk},$$

$$\Gamma_{0k}^{j}=a^{-1}\frac{da}{dt}\delta_{k}^{j}\equiv H\delta_{jk}.$$

Then, Equation (28) reads

(49) $$\begin{array}{c} p_{0}\left(\partial_{t}-2Hp^{k}\partial_{k}^{p}\right)\Omega +p^{k}\partial_{k}^{x}\Omega =\kappa^{2}\left(m^{2}a^{-2}{▵}_{p}+p^{j}p^{k}\partial_{j}^{p}\partial_{k}^{p}+3p^{k}\partial_{k}^{p}\right)\Omega , \end{array}$$

where $p_{0}=\sqrt{m^{2}+a^{2}p^{2}}$ and $p^{2}=p^{j}p^{j}$.

Let r=|p|. Then, the Formula (49) is

(50) $$\begin{array}{c} \sqrt{m^{2}+a^{2}r^{2}}\left(\partial_{t}-2Hr\partial_{r}\right)\Omega +p^{k}\partial_{k}^{x}\Omega =\kappa^{2}\left(m^{2}a^{-2}{▵}_{p}+r^{2}\partial_{r}^{2}+3r\partial_{r}\right)\Omega . \end{array}$$

On rotation invariant functions ${▵}_{p}=\partial_{r}^{2}+\frac{2}{r}\partial_{r}$. The massless diffusion is a soluble model in the spatially flat FLRW model (k=0 in Equation (17)) which will be discussed in detail in Section 5. In this case Equation (50) is

(51) $$\begin{array}{c} a\left(\partial_{t}-2Hr\partial_{r}\right)\Omega +p^{k}{\left|p\right|}^{-1}\partial_{k}^{x}\Omega =\kappa^{2}\left(r\partial_{r}^{2}+3\partial_{r}\right)\Omega . \end{array}$$

It can be seen from Equation (50) that if *H* stays bounded then the limit a→∞ of the massive diffusion is the massless diffusion. As a consequence of the rotation invariance of ${▵}_{H}$ and $p^{j}\partial_{j}=r\partial_{r}$, the diffusion operator in Equation (49) is rotationally invariant. If the initial state Ω is independent of x and rotationally invariant then the solution $\Omega_{t}$ depends only on |p|. We can calculate

(52) $$\begin{array}{c} w=p\rho^{-1}=\frac{1}{3}a^{-2}\int dpp_{0}^{-1}p^{2}\Omega_{t}{\left(\int dpp_{0}\Omega_{t}\right)}^{-1}\\ =\frac{1}{3}-\frac{1}{3}m^{2}\int dq\omega^{-1}\Omega_{t}{\left(\int dq\omega \Omega_{t}\right)}^{-1}, \end{array}$$

where we set q=ap and $\omega =\sqrt{m^{2}+q^{2}}$. Hence, $0<w\leq \frac{1}{3}$ is time-dependent. It follows from Equation (52) that w→0 when m→∞. In general, *w* could depend on x if ρ and *P* depend on x. Equation (42) with a time dependent *w* has the form

(53) $$\partial_{t}\rho +3\left(1+w_{t}\right)H\rho =3n\kappa^{2}a^{-3}.$$

The solution of Equation (53) is discussed in the next section. Note that inserting as $\Omega_{t}$ the stationary Jüttner distribution [3] of Section 5 with the temperature $\frac{\kappa^{2}}{H}$ [49] we obtain $w=\frac{1}{3}-\frac{1}{3}K$ with

(54) $$\begin{array}{c} K=\int_{0}^{\infty }dyy^{2}{\left(m^{2}+y^{2}\right)}^{-\frac{1}{2}}\exp\left(-\frac{H}{\kappa^{2}}{\left(m^{2}+y^{2}\right)}^{\frac{1}{2}}\right)\\ {\left(\int_{0}^{\infty }dyy^{2}{\left(m^{2}+y^{2}\right)}^{\frac{1}{2}}\exp\left(-\frac{H}{\kappa^{2}}{\left(m^{2}+y^{2}\right)}^{\frac{1}{2}}\right)\right)}^{-1}. \end{array}$$

## 4. Thermodynamics in an Expanding Universe

We consider a homogeneous thermodynamic system in an instantaneous equilibrium. At the fixed time for a system in a finite volume *V* we consider the first law of thermodynamics for the energy *E* with the energy density ρ (so E=ρV) where ρ=ρ(T) depends only on temperature and the entropy S=S(V,T)

(55) $$TdS=dE+PdV=\frac{\partial \rho }{\partial T}VdT+\left(P+\rho \right)dV=T\left(\frac{\partial S}{\partial T}dT+\frac{\partial S}{\partial V}dV\right),$$

(the differential is a difference between various configurations). Hence,

(56) $$\frac{\partial \rho }{\partial T}V=T\frac{\partial S}{\partial T}.$$

We assume P=wρ. Differentiating *dV* component in Equation (55) over *T* we get

(57) $$\frac{\partial S}{\partial V}+T\frac{\partial^{2}S}{\partial V\partial T}=\left(1+w\right)\frac{\partial \rho }{\partial T}.$$

Differentiating Equation (56) over *V*

(58) $$T\frac{\partial^{2}S}{\partial T\partial V}=\frac{\partial \rho }{\partial T}.$$

From Equations (55), (57) and (58) we obtain

(59) $$\frac{\partial \rho }{\partial T}=w^{-1}\left(1+w\right)\frac{\rho }{T}$$

Hence, from Equation (59)

(60) $$\rho_{st}\left(T\right)=BT^{\frac{1+w}{w}}$$

and from Equation (55)

(61) $$S_{st}=\left(1+w\right)BVT^{\frac{1}{w}},$$

where the index st refers to a fixed time. Let us consider how these equations will evolve in time. In the flat expanding metric $V=V_{0}a^{3}$ (we take $V_{0}=\frac{4\pi }{3}$ later on). Hence, if in a closed (isolated) system *S* is to be constant, with a constant *B* of Equation (60), then *T* must vary as $T=T_{0}a^{-3w}$ (a consequence of Equation (61)).

We assume that in a dissipative adiabatic process in spite of the dependence of $V=V_{0}a^{3}$ on time the entropy $S_{st}$ (61) does not change. This gives the condition

(62) $$\left(1+w\right)B\left(a\right)a^{3}T{\left(a\right)}^{\frac{1}{w}}=const$$

If we know $\rho_{st}$ (as we got it in Equation (43)) then from Equations (60) and (62) we determine T(a) for the dissipative system. In the case of the diffusion, Equation (43) together with Equations (62) and (60) define T(a) in the form (44).

Let us assume that in the diffusive system in an expanding universe the entropy is the sum

(63) $$S=S_{st}+S_{diss},$$

where $S_{st}$ is the constant from Equations (61) and (62). For the relativistic diffusion we obtain (from the equation TdS=dE+PdV, and Equations (35) and (42))

(64) $$\begin{array}{c} T\partial_{t}S_{diss}=V\left(\partial_{t}\rho \right)+V^{-1}\partial_{t}V\rho \left(1+w\right)=V3\kappa^{2}na^{-3}=3\kappa^{2}nV_{0}, \end{array}$$

where *w* may depend on time.

Then, from Equation (64)

(65) $$S_{diss}=3\kappa^{2}nV_{0}\int_{t_{0}}^{t}\frac{ds}{T(s)}+C=3nV_{0}\ln\left(T_{0}+\kappa^{2}A\right)+C,$$

where *C* is a constant. Note that in Equation (47) lnG≃−S.

The formulas above apply to time-dependent *w* with minor changes. Let

(66) $$a^{-3w_{e}\left(t,\tau \right)}\equiv \exp\left(-3\int_{\tau }^{t}w_{s}\partial_{s}\ln ads\right)$$

Then, from Equation (42)

(67) $$\begin{array}{c} \rho \left(t\right)=3na^{-3(1+w_{e}\left(t,t_{0}\right))}\left(T_{0}+\kappa^{2}\int_{t_{0}}^{t}a^{3w_{e}\left(\tau ,t_{0}\right)}d\tau \right)\equiv 3na^{-3(1+w_{e}\left(t,t_{0}\right))}B\left(a\right). \end{array}$$

We define the temperature in such a way that $S_{st}$ (62) does not change during the diffusive expansion. Then, it can be seen from Equation (67) that the formula for the temperature is

(68) $$\begin{array}{c} T\left(t,a\right)=a^{-3w_{e}\left(t,t_{0}\right)}B\left(a\right)=a^{-3w_{e}\left(t,t_{0}\right)}\left(T_{0}+\kappa^{2}\int_{t_{0}}^{t}a^{3w_{e}\left(s,t_{0}\right)}ds\right). \end{array}$$

In such a case the integration in Equation (65) gives the formula for the dissipative entropy

(69) $$S_{diss}=3nV_{0}\ln\left(T_{0}+\kappa^{2}\int_{t_{0}}^{t}a^{3w_{e}\left(s,t_{0}\right)}ds\right).$$

In fact, $S_{diss}$ is the solution of the Equation (64) $\partial_{t}S_{diss}=3\kappa^{2}nT^{-1}V_{0}$.

## 5. Solutions with the Initial Jüttner Distribution

We are unable to solve the massive Equation (28) for a general expansion *a*. In the de Sitter space a=exp(Ht), the solution with the Jüttner initial distribution (obtained in [50]) is

(70) $$\Omega_{t}=\frac{n}{8\pi }{\left(\kappa^{2}H^{-1}a\right)}^{-3}\exp\left(-\sqrt{a^{2}p^{2}+m^{2}}H\kappa^{-2}\right).$$

It looks (up to a numerical time-dependent factor) to be a static Jüttner distribution [51] in the physical momentum q=ap. In a sense at any time we have again a Jüttner-type distribution. It is expressing the fact that de Sitter space has a definite temperature (here $T=\frac{\kappa^{2}}{H}$ [49]). The diffusion equilibrates to this temperature. In the de Sitter space the relativistic diffusion (28) can be expressed as a diffusion with a time-independent generator. In fact, let us introduce new variables (a,u) with u=a|p|=ar then Equation (28) in the (t,u) variables for a rotationally invariant initial condition reads

(71) $$\begin{array}{c} \partial_{t}\Omega =\left(Hu\partial_{u}+\frac{m^{2}\kappa^{2}}{\omega }\left(\partial_{u}^{2}+\frac{2}{u}\partial_{u}\right)+\frac{\kappa^{2}}{\omega }\left(u^{2}\partial_{u}^{2}+3u\partial_{u}\right)\right)\Omega \equiv L\Omega , \end{array}$$

From Equation (71) it follows that Jüttner distribution [51] $\exp(-H\kappa^{-2}\omega )$ where $\omega =\sqrt{m^{2}+u^{2}}$ is the eigenfunction of L with the eigenvalue −3H so that $\exp\left(-3H\tau \right)\exp\left(-H\kappa^{-2}\omega \right)$ is the solution of Equation (71). For a large *u* the term proportional to the mass drops out. Then, asymptotically we obtain from Equation (71) the solution $\exp(-H\kappa^{-2}u$) of the massless diffusion (Equation (71) is still discussed in Section 7).

We restrict ourselves in this section to the soluble model of m=0. At m=0 Equation (28) reads (where r=|p|)

(72) $$\begin{array}{c} a\left(\partial_{t}-2Hr\partial_{r}\right)\Omega =\kappa^{2}\left(r\partial_{r}^{2}+3\partial_{r}\right)\Omega . \end{array}$$

This equation is exactly soluble (after a change of time) for arbitrary a(t) and general initial conditions as will be discussed in Section 7 and in the Appendix A. However, in the subsequent two sections we restrict our discussion to the solutions of this equation with an initial Jüttner distribution [51] (it has an interpretation as a relativistic Gibbs distribution [48]).

Let

ap=q.

We solve Equation (72) with the initial condition (we set the Boltzman constant $k_{B}=1$, then defining the temperature $\tilde{T_{0}}$ in Minkowski space we shall have $k_{B}{\tilde{T}}_{0}=\frac{T_{0}}{a_{0}}$)

(73) $$\Omega_{0}=\frac{n}{8\pi }T_{0}^{-3}\exp\left(-\frac{a_{0}\left|q\right|}{T_{0}}\right).$$

It is normalized as

$$\int dq\sqrt{g_{0}}\Omega_{0}=n\equiv \frac{N}{V_{0}},$$

where *N* is the number of particles, $V_{0}$ is the initial volume, so that *n* is the initial particle density. The solution of Equation (72) with the initial condition (73) at $t_{0}$ is [8,34] (we assume $a\left(t_{0}\right)=a_{0})$

(74) $$\Omega_{t}=\frac{n}{8\pi }{\left(T_{0}+\kappa^{2}A\right)}^{-3}\exp\left(-\frac{a|q|}{T_{0}+\kappa^{2}A}\right),$$

where

(75) $$A\left(t\right)=\int_{t_{0}}^{t}a\left(s\right)ds.$$

The instantaneous distribution is again of Jüttner-type. It could be considered as a solution of the maximum entropy requirement [48] for the entropy defined by the phase space distribution (see Section 6). It is normalized

(76) $$\int dq\sqrt{g_{t}}\Omega_{t}=n.$$

Note that the solution (74) in general has no limit (no normalizable equilibrium) as t→∞. The exception is the Sitter space (a=expHt) when the solution (74) becomes time-independent (up to a normalization factor) and coincides with (70) at m=0 if

$$T_{0}=H^{-1}\kappa^{2}a\left(t_{0}\right).$$

The current $N^{\mu }$ is covariantly conserved as from Equation (28)

(77) $$\nabla_{\mu }^{x}N^{\mu }=\frac{1}{\sqrt{g}}\partial_{\mu }^{x}\left(\sqrt{g}N^{\mu }\right)=a^{-3}\partial_{t}\left(a^{3}N^{0}\right)=a^{-3}\partial_{t}n=0.$$

In fact, Equation (77) holds true in general because from the diffusion Equation (28) we can show $\nabla_{\mu }^{x}N^{\mu }=0$.

The normalization (76) is a fulfillment of the conservation law

(78) $$\int_{V_{0}}dx\sqrt{-g}N^{0}=\int_{V_{0}}dxdq\sqrt{-g}\Omega =const$$

Hence, $N^{0}=\frac{n}{\sqrt{-g}}$.

For a fixed $T_{0}$ in the state (74) we calculate ρ from the phase space distribution in Equation (26) ($P=\frac{1}{3}\rho$ as a consequence of Equation (26) and $p^{\mu }p_{\mu }=0$)

(79) $$\rho =T^{00}=3na^{-4}\left(T_{0}+\kappa^{2}A\right)$$

(in agreement with the solution (43) for $w=\frac{1}{3}$). Note that a superposition

(80) $$\Omega =\int dT_{0}\nu \left(T_{0}\right)\Omega \left(T_{0}\right)$$

(with $\int dT_{0}\nu \left(T_{0}\right)=1$) will also solve the diffusion Equation (28) with the same normalization as the initial Jüttner distribution. It has the energy density

(81) $$\begin{array}{c} \rho =3na^{-4}\int dT_{0}\nu \left(T_{0}\right)\left(T_{0}+\kappa^{2}A\right)=3nCa^{-4}+3n\kappa^{2}a^{-4}A\\ =3na^{-4}\left({\tilde{T}}_{0}+\kappa^{2}A\right), \end{array}$$

where

$${\tilde{T}}_{0}=\int dT_{0}\nu \left(T_{0}\right)T_{0}.$$

## 6. Entropy Variation

In this section we suggest a covariant form of the first law of thermodynamics and statistical mechanics for a metric more general than (17). For this purpose let us introduce the inverse temperature β as a four-vector (see [2,3,52,53] and references cited there)

(82) $$\beta^{\mu }=\beta u^{\mu }.$$

Then, we consider the entropy density $\sigma^{\mu }$ as the four-vector

(83) $$\sigma^{\mu }=\sigma u^{\mu }.$$

We express the first law of thermodynamics of a moving fluid as [54]

(84) $$-\nabla_{\mu }\sigma^{\mu }=\beta_{\nu }\nabla_{\mu }T^{\mu \nu }.$$

or

$$\nabla_{\mu }\left(\beta_{\nu }T^{\mu \nu }\right)-\frac{1}{2}\left(\nabla_{\mu }\beta_{\nu }+\nabla_{\nu }\beta_{\mu }\right)T^{\mu \nu }=-\nabla_{\mu }\sigma^{\mu }.$$

Hence, if $\beta_{\mu }$ is the Killing vector then

$$\sigma^{\mu }=-\beta_{\nu }T^{\mu \nu }+k^{\mu },$$

where $\nabla_{\mu }k^{\mu }=0$. We can insert Equation (84) in the Bianchi identities (12)

$$\begin{array}{c} \partial_{t}G\int dx\sqrt{\gamma }\beta T_{0}^{0}+G\partial_{t}\int dx\sqrt{-g}\sigma^{0}+G\int dx\partial_{j}\left(\sqrt{-g}\sigma^{j}\right)\\ +\frac{1}{8\pi }\int dx\sqrt{\gamma }\beta \partial_{t}\Lambda =0. \end{array}$$

In the comoving frame $\sigma^{j}=\sigma u^{j}=0$. We define the entropy as

$$S=\int dx\sqrt{\gamma }\sigma$$

and the mass

$$M=\int dx\sqrt{\gamma }T^{00}.$$

Then, using $\sigma^{0}=u^{0}\sigma ={\left|g_{00}\right|}^{-\frac{1}{2}}\sigma$ we can rewrite the integrated Bianchi identity as

$$\begin{array}{c} \beta \partial_{t}GM+G\partial_{t}S+\frac{\beta }{8\pi }\int dx\sqrt{\gamma }\partial_{t}\Lambda =0. \end{array}$$

It is interesting to note that if we apply this equation (with Λ=0) to a system with the horizon 2GM, the Hawking temperature $T={\left(8\pi GM\right)}^{-1}$ and the Hawking formula for the entropy $S=\frac{4\pi {\left(2GM\right)}^{2}}{4G}$ then we get as a conclusion $G≃M^{-\frac{2}{3}}$.

For a diffusing fluid from Equation (35) we obtain

(85) $$3\kappa^{2}\beta^{\nu }N_{\nu }=-\nabla_{\mu }\sigma^{\mu }.$$

In the comoving frame (from Equation (85))

(86) $${\left(-g\right)}^{-\frac{1}{2}}\partial_{t}\left(\sigma^{0}\sqrt{-g}\right)=3\kappa^{2}\beta_{0}N^{0}.$$

With the temperature (44) Equation (86) reads

(87) $$\partial_{t}\left(\sigma^{0}a^{3}\right)=\partial_{t}\ln\left(T_{0}+\kappa^{2}\int_{t_{0}}^{t}a^{3w}\right).$$

Hence,

(88) $$\sigma^{0}a^{3}=3n\ln\left(T_{0}+\kappa^{2}\int_{t_{0}}^{t}a^{3w}\right)+C.$$

(we assume that all variables depend only on time). We integrate over an expanding volume $V=V_{0}\sqrt{g}$ (we assume the isotropic expansion |x(t)|=a(t), a(0)=1, $V_{0}=\frac{4\pi }{3}$) then

(89) $$S=\int dx\sqrt{g}\sigma =\sigma \sqrt{g}V_{0}=\frac{4\pi }{3}\sigma a^{3}.$$

Hence, from Equations (84) and (85) we recover our result (69).

We still rewrite Equation (84) using $\nabla_{\mu }u^{\mu }=3H$ (for FLRW metric (17))

(90) $$\nabla_{\mu }\sigma^{\mu }=u^{\mu }\nabla_{\mu }\sigma +\sigma \nabla_{\mu }u^{\mu }=u^{\mu }\nabla_{\mu }\sigma +3H\sigma .$$

In the comoving frame u=(1,0) we obtain from Equation (84) and the energy-momentum tensor of an ideal fluid the fluid continuity equation

(91) $$\partial_{t}\rho +3H\left(\rho +\tilde{P}\right)=T\partial_{t}\sigma ,$$

where $\tilde{P}=P-\sigma T$ and $\rho +\tilde{P}$ is the entalpy density. Equation (91) expressed in terms of the entropy (89) gives the first law of thermodynamics (54) (with $V=\frac{4\pi }{3}a^{3})$

(92) $$\partial_{t}\rho +3H\left(\rho +P\right)=\frac{3}{4\pi a^{3}}T\partial_{t}S.$$

Inserting the Bianchi identity (19) in Equation (92), we express it in the form

(93) $$8\pi \rho \partial_{t}G+8\pi TG\left(\nabla_{t}\sigma +3H\sigma \right)+\partial_{t}\Lambda =0.$$

or in terms of the entropy *S* (89),

(94) $$\begin{array}{c} a^{-3}GT\frac{dS}{dt}=-\frac{4\pi }{3}\frac{dG}{dt}\rho -\frac{1}{6}\frac{d\Lambda }{dt}. \end{array}$$

For a diffusing system of particles having a phase space distribution Ω we can calculate the statistical physics (microscopic) entropy $\sigma^{\mu }$ from the formula [3,55]

(95) $$\sigma^{\mu }=-\sqrt{g}\int dpp_{0}^{-1}p^{\mu }\Omega \ln\Omega =\sigma u^{\mu }.$$

In the comoving frame $\sigma =\sigma^{0}$. Then, from Equations (74) and (95)

(96) $$\begin{array}{c} \sigma =-\sqrt{g}\int dp\Omega \ln\Omega =-\int dq\Omega \left(-{\left(T_{0}+\kappa^{2}A\right)}^{-1}\left|q\right|-3\ln\left(T_{0}+\kappa^{2}A\right)+\ln n\right)\\ =3na^{-3}\ln\left(T_{0}+\kappa^{2}A\right)-a^{-3}\left(n\ln n-3n\right). \end{array}$$

Then

(97) $$S=\frac{4\pi }{3}sa^{3}=4\pi n\ln\left(T_{0}+\kappa^{2}A\right)-\frac{4\pi }{3}\left(n\ln n-3n\right).$$

Hence, in Equation (65) (with $V_{0}=\frac{4\pi }{3}$) we have $C=\frac{4\pi }{3}\left(-n\ln n+3n\right)$. The statistical and thermodynamic entropies coincide. When expressed by the temperature $S=4\pi n\ln(a^{3w}T)$, the result for the entropy resembles the Sackura–Tetrode formula for a gas of free non-relativistic particles.

For massive particles in the de Sitter space the Formula (95) with the solution (70) gives

(98) S≃Kt

It can be seen that such a result also follows from Equation (97) as $\ln(T_{0}+A)≃t$. For a power-law expansion we obtain a slower growth of entropy S≃lnt at large time.

We have derived the result for the entropy of the gas of diffusing particles first from thermodynamics knowing the formula for the energy density. Then, this result has been obtained from a microscopic Gibbs-type distribution as a solution of the diffusion equation. Clearly, for other solutions of the diffusion equation we would get from Equation (95) another formula for the entropy of this distribution (eventually asymptotically as t→∞ we could approach (97)).

## 7. Solution of Einstein and Diffusion Equations

We can get a general solution of the massless diffusion Equation (72) in a general spatially flat expanding metric because after a change of variables it can be reduced to the equation in the Minkowski space time solved in [34,56]. Let in Equation (72) $v=a^{2}r=a^{2}\left|p\right|$. We introduce a new time η

(99) $$\kappa^{2}\eta =T_{0}+\kappa^{2}\int_{t_{0}}^{t}a\left(s\right)ds$$

The variable lnη≃S related to the entropy *S* in Equation (65) and to the temperature by Equation (44) could be considered as a thermal time [57] in general relativity (in fact, in the state (74) the relation $\frac{d}{dt}=\frac{1}{T}\frac{d}{d\eta }$ of ref. [58] holds true with the temperature *T* (44) although the state (74) is not stationary). In the variables (η,v), Equation (72) reads

(100) $$\begin{array}{c} \partial_{\eta }\Omega +a^{-2}p^{k}{\left|p\right|}^{-1}\partial_{k}^{x}\Omega =\kappa^{2}\left(v\partial_{v}^{2}+3\partial_{v}\right)\Omega . \end{array}$$

The momentum part of Equation (100) becomes time-independent. For further purposes we consider a slightly more general equation

(101) $$\begin{array}{c} \partial_{\eta }\Omega +a^{-2}p^{k}{\left|p\right|}^{-1}\partial_{k}^{x}\Omega =hv\partial_{v}\Omega +\kappa^{2}\left(v\partial_{v}^{2}+3\partial_{v}\right)\Omega =L\Omega . \end{array}$$

In the variable $y=-\kappa^{-2}hv$, Laguerre polynomials [59] are solutions of the eigenvalue equation

(102) $$LL_{k}^{\left(2\right)}\left(y\right)=khL_{k}^{\left(2\right)}\left(y\right).$$

It follows that $\exp\left(kh\eta \right)L_{k}^{\left(2\right)}\left(y\right)$ are solutions of the diffusion Equation (101). Hence, an arbitrary sum of these functions is a solution. It is useful to apply the known formulas for the summation of Laguerre polynomials [59]

(103) $$\sum_{k=0}^{\infty }s^{k}L_{k}^{\left(\alpha \right)}\left(y\right)={\left(1-s\right)}^{-\alpha -1}\exp\left(-\frac{sy}{1-s}\right)$$

and

(104) $$\begin{array}{c} \sum_{k=0}^{\infty }n!\Gamma {\left(k+\alpha +1\right)}^{-1}L_{k}^{\left(\alpha \right)}\left(x\right)L_{k}^{\left(\alpha \right)}\left(y\right)s^{k}\\ ={\left(1-s\right)}^{-1}{\left(xys\right)}^{-\frac{1}{2}\alpha }\exp\left(-s{\left(1-s\right)}^{-1}\left(x+y\right)\right)I_{\alpha }\left(2{\left(1-s\right)}^{-1}\sqrt{xys}\right), \end{array}$$

where $I_{\alpha }$ is the Bessel function. Using the orthogonality property of Laguerre polynomials

(105) $$n!\Gamma {\left(k+\alpha +1\right)}^{-1}\int_{0}^{\infty }dxx^{\alpha }L_{n}^{\left(\alpha \right)}\left(x\right)L_{m}^{\left(\alpha \right)}\left(x\right)=\delta_{nm}$$

we can obtain a solution of the diffusion equation with any initial condition.

Using Equations (102)–(105) we can generate solutions of Equation (101). Then, taking the limit h→0 we obtain a general solution of Equation (100). From Equation (103) we obtain in the limit h→0 (up to a normalization) the solution (74)

(106) $$\Omega_{\eta }\left(v\right)=\eta^{-3}\exp\left(-\kappa^{-2}\frac{v}{\eta }\right)$$

From Equation (104) we obtain an evolution kernel for Equation (101)

(107) $$\begin{array}{c} \Omega_{\eta }\left(v,v^{\prime }\right)=K{\left(vv^{\prime }\eta \right)}^{-1}\exp\left(-\frac{v+v^{\prime }}{\kappa^{2}\eta }\right)I_{2}\left(\frac{2\sqrt{vv^{\prime }}}{\kappa^{2}\eta }\right), \end{array}$$

where *K* is a normalization constant such that when η→0 then $\Omega_{\eta }\left(v,v^{\prime }\right)\to \delta \left(v-v^{\prime }\right)$

The solution of the space-time dependent diffusion equation (with the initial condition $\Omega (x,p|p{|}^{-1},|p|))$ reads

(108) $$\begin{array}{c} \Omega_{\eta }{\left(x,p|p\right|}^{-1},|p|)=E[\Omega (x-{p|p|}^{-1}{\tau ,p|p|}^{-1},v_{\eta })]\\ =\int_{0}^{\infty }dv^{\prime }{\Omega (x-p|p|}^{-1}{\tau ,p|p|}^{-1},v^{\prime })\Omega_{\eta }\left(v,v^{\prime }\right), \end{array}$$

where E[…] denotes the expectation value over the stochastic process $v_{\eta }$ generated by the diffusion (101) (see Appendix A) and $\tau =\int dta^{-1}$ is the conformal time, $\Omega_{\eta }\left(v,v^{\prime }\right)$ is the evolution kernel (107) (the diffusion transition function).

The phase space solution (108) could be used for a calculation of space and momentum evolution of a cluster of particles. However, it is not simple to apply Equations (26) and (27) for analytic calculations of ${\hat{T}}^{\mu \nu }$ and the current $N^{\mu }$ of Equations (26) and (27). The solution (108) is heavily dependent on the initial condition and it is not so explicit concerning its dependence on the cosmic time *t*. For this reason, let us discuss some simple solutions based on the equations for ρ and *a*. If G=const then the Bianchi identity (19) (or (45)), the expression of the energy-momentum non-conservation (42) and the first Friedmann Equation (23) constitute a closed system of first order differential equations for Λ,ρ,a with the unique solution under given initial conditions (in particular, we may assume for Λ zero or non-zero initial condition). If we admit *G* to depend on time then in order to have the unique solution we must choose one of the functions a,ρ,Λ or *G*.

We can look for a solution of the diffusion Equation (28) as a part of a more ambitious cosmological program of a description of the formation and time evolution of galaxies in the universe [60] by means of their phase space distribution $\Omega_{t}\left(x,p\right)$. Such a project requires a solution of Einstein Equation (2) together with the solution of the diffusion Equation (28) (for other applications of the diffusion’s phase space distribution see [24,35,36]). We may begin with a simple task of solving the diffusion equation with a fixed metric $g_{\mu \nu }$ which eventually can also solve the Einstein Equation (2). We define ρ and *P* by Equation (26) (as a function of *a*). Then, the Friedmann Equation (23) and the Bianchi identities determine two of the three functions a,G,Λ.

As a simple example we consider G=const and look for a (asymptotic) power-law solution

(109) $$a\left(t\right)=a_{1}t^{\alpha }.$$

From Equation (43)

(110) $$\rho_{t}=Ct^{-3\alpha (1+w)}+At^{-3\alpha +1}.$$

Then, from the Friedmann Equation (23) we obtain α=1 (coasting solution [41]) and (C=0 in Equation (110) or $w=-\frac{1}{3}$)

(111) $$\rho \left(t\right)=At^{-2}$$

(112) $$\Lambda =Bt^{-2}$$

Equations (109)–(112) (with α=1 in Equation (109) and C=0 in Equation (110)) deliver a particular exact solution of the Equations (19), (23) and (42) of the diffusive gas (Equation (42) is a consequence of the diffusion Equation (28)). It can be seen from the derivation that under the assumption G=const the solution (109) with α=1 is the asymptotic power-law solution of Equations (19), (23) and (42) (there may be an asymptotic exponential solution of these equations if the initial condition for Λ is a positive number).

With ρ and Λ of Equations (109)–(112) we still must determine the phase space distribution Ω as the solution of the diffusion Equation (28). There may be various solutions of the diffusion Equation (28) (with different initial conditions) leading to the energy-momentum and the current satisfying Equations (19), (23) and (42) (with the solution (109)–(112)). A particular solution of the diffusion equation which leads to Einstein equations with $a\left(t\right)=a_{1}t$, ρ (111) and Λ (112) is the solution of Equation (72) for massless particles

(113) $$\Omega_{t}=t^{-6}\exp(-2\kappa^{-2}|p|).$$

This solution follows from Equation (74) with a special choice of parameters $T_{0}$, $t_{0},a_{1}$ in such a way that

$$\kappa^{2}\eta =T_{0}+\kappa^{2}\int_{t_{0}}^{t}a_{1}sds=\frac{\kappa^{2}}{2}t^{2}.$$

We could find a solution of Equations (19), (23) and (45) with an arbitrary α in Equation (109), if *G* is time-dependent. If the solution for evolution equations is searched in the form of a power-law then there are power-law (in cosmic time) components for *a* ρ, *G* and Λ.

The coasting evolution $a\left(t\right)=a_{1}t$ is exceptionally simple. In particular, with a rotation invariant space independent initial condition, if we introduce the conformal time $\tau =\int dta^{-1}=\ln t$, then Equation (72) can be expressed as (with r=|p|)

(114) $$\partial_{\tau }\Omega =2r\partial_{r}\Omega +\kappa^{2}\left(r\partial_{r}^{2}+3\partial_{r}\right)\Omega =L\Omega .$$

This is a special case of Equation (101) (h=2). The operator L in the variable $y=-2\kappa^{-2}r$ is related to the operator defining the Laguerre polynomials $L_{k}^{\left(\alpha \right)}$ with α=2. In fact,

(115) $$LL_{k}^{\left(2\right)}\left(y\right)=2kL_{k}^{\left(2\right)}\left(y\right).$$

It follows that the functions $\exp\left(2k\tau \right)L_{k}^{\left(2\right)}$ are solutions of Equation (114). Hence, also the sum of these functions is a solution. Using the summation Formula (103) for the Laguerre polynomials we obtain a normalizable solution of the diffusion Equation (114) (τ is the conformal time)

(116) $$\Omega_{\tau }={\left(\exp\left(2\tau \right)-1\right)}^{-3}\exp\left(-\frac{2\exp(2\tau )}{\exp(2\tau )-1}\kappa^{-2}r\right).$$

The limit τ→∞ of Equation (116) is also a solution of Equation (114). It coincides with (113).

Using Equation (26) we can calculate the energy density for the solution (116). It is (in agreement with Equation (79))

(117) $$\rho_{t}=At^{-2}+Ct^{-4}$$

with certain constants *A* and *C*.

We can obtain a general solution of Equation (114) with an arbitrary initial condition Ω. For this purpose we expand the initial Ω in Laguerre polynomials $\Omega =\sum c_{k}L_{k}^{\left(2\right)}$. Then, the solution of Equation (114) expressed as an infinite sum is

(118) $$\Omega_{\tau }\left(y\right)=\sum_{k=0}^{\infty }c_{k}\exp\left(2k\tau \right)L_{k}^{\left(2\right)}\left(y\right).$$

The solution of the space-time diffusion with the initial condition $\Omega (x,p|p{|}^{-1},|p|)$ is (see Appendix A)

(119) $$\begin{array}{c} \Omega_{\tau }{\left(x,p|p\right|}^{-1},|p|)=\int_{0}^{\infty }dr^{\prime }{\Omega (x-p|p|}^{-1}{\tau ,p|p|}^{-1},r)\Omega_{\eta }\left(r,r^{\prime }\right), \end{array}$$

where r=|p|, τ is the conformal time, $\Omega_{\eta }\left(r,r^{\prime }\right)$ is the evolution kernel of the diffusion operator (114) (the transition function for the diffusion (114) defined in Equation (107)). The elementary dependence of the phase space distribution on x is a consequence of Equation (49) as it follows from this equation that ${p|p|}^{-1}$ does not change during the diffusion (see Appendix A and [34,56]).

For the next simple example of a solution of a diffusion Equation (28) in the cosmic time, let us consider an exponential expansion a=exp(tH) (H=const). Equation (28) can be expressed as a diffusion with a time-independent generator. Let u=ra then Equation (50) takes the form

(120) $$\begin{array}{c} \partial_{t}\Omega =Hu\partial_{u}\Omega +\kappa^{2}{\left(m^{2}+u^{2}\right)}^{-\frac{1}{2}}\left(m^{2}\left(\partial_{u}^{2}+\frac{2}{u}\partial_{u}\right)+u^{2}\partial_{u}^{2}+3u\partial_{u}\right)\Omega . \end{array}$$

For large *u* the *m*-dependent terms are of the order $mu^{-1}$ in Equation (120). Hence, the solution of Equation (120) for a large *u* should not depend on *m*. Neglecting the $mu^{-1}$ terms we obtain the equation

(121) $$\begin{array}{c} \partial_{t}\Omega =Hu\partial_{u}\Omega +\kappa^{2}\left(u\partial_{u}^{2}+3\partial_{u}\right)\Omega \equiv L\Omega . \end{array}$$

We have obtained Equation (101) but now in the cosmic time. Introducing the variable $y=-\kappa^{-2}Hu$ we can see that Laguerre polynomials are solutions of the eigenvalue equation

(122) $$LL_{k}^{\left(2\right)}\left(y\right)=kHL_{k}^{\left(2\right)}\left(y\right).$$

We may take the sum of Laguerre polynomials using the generating function (103) in order to obtain a normalizable solution of the diffusion Equation (121)

(123) $$\begin{array}{c} \Omega_{t}\left(p\right)={\left(\exp\left(Ht\right)-1\right)}^{-3}\\ \exp\left(-H\kappa^{-2}\left|p\right|\exp\left(2Ht\right){\left(\exp\left(Ht\right)-1\right)}^{-1}\right) \end{array}$$

(we skip the normalization constant). The limit t→∞ of (123) is also the solution of Equation (121). It coincides with the exact solution (70) of Equation (121) in the $mu^{-1}$ expansion. As in the general expansion a(t) (108) and in the case of the coasting expansion we can obtain the general solution of the massless equation expanding the initial condition (the momentum part) $\Omega (x,p|p{|}^{-1},|p|)$ in Laguerre polynomials by means of the orthogonality Formula (105) (another method is to apply the propagation kernel $\Omega_{\eta }\left(v,v^{\prime }\right)$ of Equation (107)). We can also express the evolution kernel $\exp\left(\eta L\right)\left(r,r^{\prime }\right)$ by Laguerre polynomials using Equation (104). Then, the general solution of Equation (121) can be expressed in the form similar to (108). A probabilistic expression of the solution (108) as well as (119) is discussed in Appendix A.

For the next example of a particular solution in the cosmic time of the diffusion equation, let us consider Equation (28) in a flat expanding metric with a large mass *m* (we neglect the terms of order $\frac{1}{m^{2}}$ and $\frac{\kappa^{2}}{m}$ in Equation (49)). Then, this equation reads

(124) $$\begin{array}{c} \left(\partial_{t}-2H\left(t\right)p^{k}\partial_{k}^{p}\right)\Omega +m^{-1}p^{k}\partial_{k}^{x}\Omega =\kappa^{2}ma^{-2}{▵}_{p}\Omega . \end{array}$$

Equation (124) describes a diffusion in momentum space with a time-dependent diffusion constant. Its solution can be expressed by the Brownian motion. This is the Gaussian stochastic process defined by the variance (E[…] denotes the expectation value with respect of the Brownian motion)

(125) $$E\left[b^{j}\left(t\right)b^{k}\left(s\right)\right]=min\left(t,s\right)\delta^{jk}.$$

Then, the solution of Equation (124) with an initial condition Ω(x,p) at $t_{0}$ can be expressed as (written for the zero initial time)

(126) $$\Omega_{t}\left(x,p\right))=E\left[\Omega \left(x-tm^{-1}p,p\left(t\right)\right)\right],$$

where

(127) $$\begin{array}{c} p\left(t\right)=\exp\left(\int_{t_{0}}^{t}ds2H\left(s\right)\right)p+\kappa \sqrt{2m}\int_{t_{0}}^{t}\exp\left(\int_{s}^{t}ds^{\prime }2H\left(s^{\prime }\right)\right)a{\left(s\right)}^{-1}db\left(s\right). \end{array}$$

We can obtain explicit integral formulas for the phase space evolution of the solution (127) if we represent the initial condition Ω(x,p) by means of its Fourier transform. Then, the expectation value over the Brownian motion in Equation (126) can be calculated, e.g., the expectation value of its Fourier factor is

(128) $$\begin{array}{c} E[\exp\left(\kappa \sqrt{2m}y\int_{t_{0}}^{t}\exp\int_{s}^{t}ds^{\prime }2H\left(s^{\prime }\right))a{\left(s\right)}^{-1}db\left(s\right)\right)]\\ =\exp\left(\exp\left(m\kappa^{2}y^{2}\int_{t_{0}}^{t}ds\exp\int_{s}^{t}ds^{\prime }4H\left(s^{\prime }\right))a{\left(s\right)}^{-2}\right)\right). \end{array}$$

With our result on the solution (123) of the diffusion Equation (121) for an exponential expansion we can approach the problem of the solution of Einstein equations assuming that the presumed exponential expansion is an exact solution (as discussed earlier in order to obtain the unique solution of Einstein equations for (a,ρ,G,Λ), one of these functions must be chosen deliberately; here we discuss the choice of *a* as in (121))

(129) $$a\left(t\right)=a_{0}\exp\left(Ht\right)$$

For the scale factor a(t) (129) we can calculate $\rho_{t}$ using Equation (43) with the approximate constant value of $w_{t}$ from Equation (52) (and its approximation at the end of Section 3, w≃0 for m→∞)

(130) $$\rho_{t}=A\exp\left(-3H\left(1+w\right)t\right)+B\exp\left(-3Ht\right).$$

The Friedmann Equation (24) gives

(131) $$3H^{2}=\Lambda -4\pi G\left(1+3w\right)\rho .$$

Inserting Λ (131) in Bianchi identity (19) we obtain

(132) $$\frac{3}{2}\left(1+w\right)\rho \partial_{t}G+\frac{1}{2}\left(1+3w\right)\partial_{t}\rho G+3n\kappa^{2}Ga^{-3}=0$$

We set ρ of Equation (130) in Equation (132) to obtain a linear equation for *G* with the solution

(133) $$\begin{array}{c} G\left(t\right)=G\left(t_{0}\right){\left(A\exp\left(-3H\left(1+w\right)t\right)+B\exp\left(-3Ht\right)\right)}^{-\nu }\\ {\left(A\exp\left(-3H\left(1+w\right)t_{0}\right)+B\exp\left(-3Ht_{0}\right)\right)}^{\nu }\\ {\left(A+B\exp\left(3wHt\right)\right)}^{-\beta }{\left(A+B\exp\left(3Hwt_{0}\right)\right)}^{\beta }, \end{array}$$

where

(134) $$\nu =\frac{1+3w}{3(1+w)}$$

and

(135) $$\beta =2\kappa^{2}na_{0}^{-3}{\left(3Hw\left(1+w\right)\right)}^{-1}.$$

For a large *t* and w≥0 it follows from Equation (133) that

(136) G≃exp(3Ht(ν−wβ))

and

(137) Gρ≃exp(−3H((1−ν)+wβ)t)

Λ can be calculated from Equation (131). Λ is decreasing in time to its de Sitter value $3H^{2}$. It is noteworthy that an exact exponential expansion leads to a growing Newton constant (136). The diffusion responsible for a varying *G* modifies ρ of Equation (130) to Gρ (137) entering the Friedman Equation (23).

Summarizing, in comparison to standard Friedmann equations (without diffusion) we have an additional equation relating ∇T with a conserved current *N*. This equation can be applied to express ρ by *a*. Then, the Bianchi identity is relating *G* to Λ.

The Equations (24), (42) and (44) can be solved for particles with m>0. Then, the index *w* is time dependent. In such a case we do not expect exact power-law solutions. However, the solutions with constant *w* can supply good approximations (e.g., small w≃0 for heavy particles and, $w≃\frac{1}{3}$ for light particles).

## 8. Summary

We studied the thermodynamics and statistical mechanics of a gas diffusing in the Einstein expanding universe. The model can find applications to a gas of galaxies expanding in a medium of an unknown origin. The thermodynamics are described by the energy density, pressure, entropy and temperature. In the diffusive model the covariant derivative of the energy-momentum tensor is proportional to a conserved particle current. The current allows a definition of the energy-momentum tensor of an ideal fluid. For such systems we derive equations relating the energy-momentum tensor to entropy and temperature. The thermodynamics of the model are determined by the covariant derivative of the energy-momentum tensor. The statistical mechanics results from the phase space distribution of diffusing particles. The phase space distribution can be obtained as a solution of the diffusion equation. The statistical mechanics of a non-relativistic diffusive motion on a static Riemannian manifold is well-known. We extend the statistical description to a relativistic diffusion on an expanding universe. We have obtained the general solution of the relativistic diffusion equation for massless particles. The massless equation is the limit of the massive diffusion equation for a large time. Most interesting for a statistical description are the solutions with Jüttner initial distribution which can be obtained from the maximum entropy requirement. The solutions determine the energy-momentum tensor and the particle current of an ideal fluid. The Einstein equations require that the covariant derivative of the total energy-momentum on the rhs of Einstein equations is zero. We express this energy-momentum tensor by the cosmological term and the energy-momentum tensor of an ideal fluid multiplied by the Newton “constant”. In this way a variation of the cosmological constants can be expressed by a change of entropy. We show that the thermodynamic entropy coincides with the microscopic entropy defined by the phase space distribution resulting as the solution of the diffusion equation with the Jüttner initial distribution. We show that the entropy can be considered as a distinguished time parameter which makes the coefficients in the diffusion equation in an expanding universe time-independent. 

## Data Availability

No new data were created or analyzed in this study. Data sharing is not applicable to this article.

## Appendix A

We begin with a simple stochastic description of the diffusion for the coasting expansion which is an asymptotic solution of diffusive Einstein equations. The solution of the diffusion Equation (49) for m=0 and a=t can be expressed by the solution of the stochastic equations in the conformal time (τ=lnt) in the Stratonovitch form [61]

(A1) $$dx=-{p|p|}^{-1}d\tau ,$$

(A2) $$dp\left(\tau \right)=3\kappa^{2}{p|p|}^{-1}d\tau +2pd\tau -\frac{1}{2}\kappa^{2}{p|p|}^{-1}d\tau +\sqrt{2}\kappa p{\left|p\right|}^{-\frac{1}{2}}\circ db\left(\tau \right).$$

The Ito and Stratonovitch (denoted by the circle) differentials [61] are related as

$${\left|p\right|}^{-\frac{1}{2}}p^{k}\circ db={\left|p\right|}^{-\frac{1}{2}}p^{k}db+\partial_{j}{\left(|p\right|}^{-\frac{1}{2}}p^{k})dp^{j}db$$

From Equation (A2) we can obtain (by differentiation) that $d(p|p{|}^{-1})=0$. Hence, ${p|p|}^{-1}$ is equal to its initial value. We can also calculate using Equation (A2)

(A3) $$d|p|=3\kappa^{2}+2\kappa^{2}\left|p\right|d\tau -\frac{1}{2}\kappa^{2}d\tau +\kappa \sqrt{2|p|}\circ db.$$

Hence, it follows that with r=|p| the diffusion Equation (51) for the coasting expansion a=t is satisfied by

(A4) $$\Omega_{\tau }\left(|p|\right)=E[\Omega (|p_{\tau }|)].$$

From the stochastic Equations (A1)–(A4) and the consequence of Equation (A2) that ${p|p|}^{-1}$ is time independent it follows that the solution of Equation (49) for m=0 with a spatial dependence of Ω can be expressed as

(A5) $$\Omega_{\tau }{\left(x,p|p\right|}^{-1},|p|))=E[\Omega (x-{p|p|}^{-1}{\tau ,p|p|}^{-1},|p_{\tau }|)].$$

The stochastic process $r_{\tau }=\left|p_{\tau }\right|$ is the solution either of the Stratonovitch Equation (A3) or the Ito equation

(A6) $$dr=3\kappa^{2}d\tau +2rd\tau +\sqrt{2r}db$$

There are minor changes in the derivation of the solution of Equation (121) for the exponential expansion. The Formula (A5) still applies in the form

(A7) $$\Omega_{t}{\left(x,p|p\right|}^{-1},|p|))=E[\Omega (x-{p|p|}^{-1}{\tau ,p|p|}^{-1},u_{t})],$$

where $u_{t}$ is the solution of the Ito equation

(A8) $$du=Hudt+3\kappa^{2}dt+\kappa \sqrt{2u}db\left(t\right).$$

For an alternative study of the relativistic diffusion (28) it is useful to derive its connection with a soluble model of quantum mechanics of an anharmonic oscillator with the Hamiltonian [62]

(A9) $$H=-\frac{1}{2}\partial_{x}^{2}+\frac{1}{2}mx^{2}+gx^{-2}.$$

The eigenvalues of this Hamiltonian are $\epsilon_{n}=2mn+m\left(\alpha +1\right)$ where

$$\alpha =\frac{1}{2}\sqrt{1+8g}.$$

The eigenfunctions can be expressed by Laguerre polynomials of order *n*. Let $\psi_{0}$ be the lowest eigenstate of *H* then

(A10) $$L=\psi_{0}^{-1}H\psi_{0}=-r\partial_{r}^{2}-\left(\alpha +1\right)\partial_{r}+2mr\partial_{r}$$

where $r=\frac{1}{2}x^{2}$. In Equation (A10) (with α=2) we obtain the diffusion generator of Equation (101).

Returning to the general massless Equation (51) in the time variable η (101) and $v=a^{2}r$ we have

(A11) $$\begin{array}{c} \partial_{\eta }\Omega +a^{-2}p^{k}{\left|p\right|}^{-1}\partial_{k}^{x}\Omega =\kappa^{2}\left(v\partial_{v}^{2}+3\partial_{v}\right)\Omega . \end{array}$$

The original stochastic process for x and p reads

(A12) $$dx=-{p|p|}^{-1}d\tau$$

where τ is the conformal time $\tau =\int a^{-1}$ and

(A13) $$dp\left(\eta \right)=3\kappa^{2}{p|p|}^{-1}d\eta +hpd\eta -\frac{1}{2}\kappa^{2}{p|p|}^{-1}d\eta +\sqrt{2}\kappa p{\left|p\right|}^{-\frac{1}{2}}\circ db\left(\eta \right),$$

where η is defined in Equation (99). It follows that the solution of the diffusion Equation (51) for an arbitrary evolution a(t) can be expressed by Equation (108).
